# A Two-Tiered Mechanism Enables Localized Cdc42 Signaling during Enterocyte Polarization

**DOI:** 10.1128/MCB.00547-16

**Published:** 2017-03-17

**Authors:** Lucas J. M. Bruurs, Susan Zwakenberg, Mirjam C. van der Net, Fried J. Zwartkruis, Johannes L. Bos

**Affiliations:** Molecular Cancer Research and Cancer Genomics Netherlands, Center for Molecular Medicine, University Medical Center Utrecht, Utrecht, The Netherlands

**Keywords:** Cdc42, GTPase signaling, cell polarity, cell signaling, polarized epithelia

## Abstract

Signaling by the small GTPase Cdc42 governs a diverse set of cellular processes that contribute to tissue morphogenesis. Since these processes often require highly localized signaling, Cdc42 activity must be clustered in order to prevent ectopic signaling. During cell polarization, apical Cdc42 signaling directs the positioning of the nascent apical membrane. However, the molecular mechanisms that drive Cdc42 clustering during polarity establishment are largely unknown. Here, we demonstrate that during cell polarization localized Cdc42 signaling is enabled via activity-dependent control of Cdc42 mobility. By performing photoconversion experiments, we show that inactive Cdc42-GDP is 30-fold more mobile than active Cdc42-GTP. This switch in apical mobility originates from a dual mechanism involving RhoGDI-mediated membrane dissociation of Cdc42-GDP and Tuba-mediated immobilization of Cdc42-GTP. Interference with either mechanism affects Cdc42 clustering and as a consequence impairs Cdc42-mediated apical membrane clustering. We therefore identify a molecular network, comprised of Cdc42, the guanine nucleotide exchange factor (GEF) Tuba, and RhoGDI, that enables differential diffusion of inactive and active Cdc42 and is required to establish localized Cdc42 signaling during enterocyte polarization.

## INTRODUCTION

Cdc42 signaling controls multiple aspects of tissue morphogenesis by regulating processes such as junction formation and cell polarization (1). As these processes are often localized to a specific part of the cell, Cdc42 activity must be confined in a local cluster to prevent ectopic signaling. During cell polarization, Cdc42 is recruited to the nascent apical membrane where it governs the positioning of the apical domain (1, 2). As a consequence, loss of Cdc42 results in the formation of multiple apical domains in a single-cell model for enterocyte polarization and in the formation of multiple lumina in three-dimensional (3D) cyst cultures (3, 4). In order to restrict apical membrane formation to a single site, Cdc42 itself must be confined into a single cluster. We previously demonstrated that an inability to restrict Cdc42 localization results in the formation of an enlarged apical membrane and consequently in the formation of an aberrantly shaped lumen (3). Despite the importance of Cdc42 clustering, little is known about the molecular mechanisms that drive Cdc42 cluster formation during cell polarization.

Cdc42 dynamics have been extensively studied in the context of Saccharomyces cerevisiae cell polarization (5–10). Clustered Cdc42 activity is required for yeast cell polarization, but it also ensures that only one polarization site is established (5, 11). As a consequence, interference with Cdc42 mobility affects the apical accumulation of Cdc42 and may give rise to yeast cells with multiple bud sites (5, 9, 12). These findings in yeast therefore indicate that control over Cdc42 dynamics is crucial for Cdc42 clustering and signaling. However, it remains unclear whether regulation of Cdc42 mobility is of similar relevance during mammalian cell polarization.

Formation of a single cluster of Cdc42 activity from an initially homogenous situation can occur by means of a Turing-type reaction-diffusion mechanism (13). The dynamics of Cdc42 clustering during yeast cell polarization have been described in mathematical models according to this mechanism (14). In these models, Cdc42-GTP functions as a typical “activator” that, by recruiting a guanine nucleotide exchange factor (GEF)/effector complex that is of limited availability, is able to promote its own synthesis, using Cdc42-GDP as a substrate. A key requirement for this activator-substrate model is differential diffusion of the reactive components, with the activator, Cdc42-GTP, typically having a low mobility (14, 15).

Here, we show that during enterocyte cell polarization, Cdc42 mobility is regulated in an activity-dependent manner, with inactive Cdc42-GDP being highly mobile and active Cdc42-GTP being relatively immobile. This striking difference in mobility is enabled via selective membrane dissociation of Cdc42-GDP by RhoGDI (where GDI is guanine nucleotide dissociation inhibitor) and immobilization of Cdc42-GTP by the Cdc42-specific GEF Tuba. We show that this switch-like diffusive behavior is critical for Cdc42 function as interfering with either mechanism impedes spatial confinement of Cdc42 signaling, resulting in an inability to establish a clustered apical membrane.

## RESULTS

Previously, we reported that during enterocyte polarization Cdc42 is apically concentrated to ensure clustering of the nascent apical membrane (3). To study the mechanisms that drive Cdc42 clustering in this process, we made use of Ls174T:W4 cells (W4 cells). These cells can polarize in the absence of cell-cell contacts by means of forced LKB1 activation, prompted by doxycycline-induced STRAD expression (16). To determine the mobility of Cdc42, we expressed it, tagged with the photoconvertible Dendra fluorophore, in polarized Ls174T:W4 cells and locally converted Dendra-Cdc42 in the apical brush border. Subsequent loss of red signal from the converted region was used to determine an eviction half-life, which reflects Cdc42's mobility at the apical plasma membrane.

**FIG 1:**
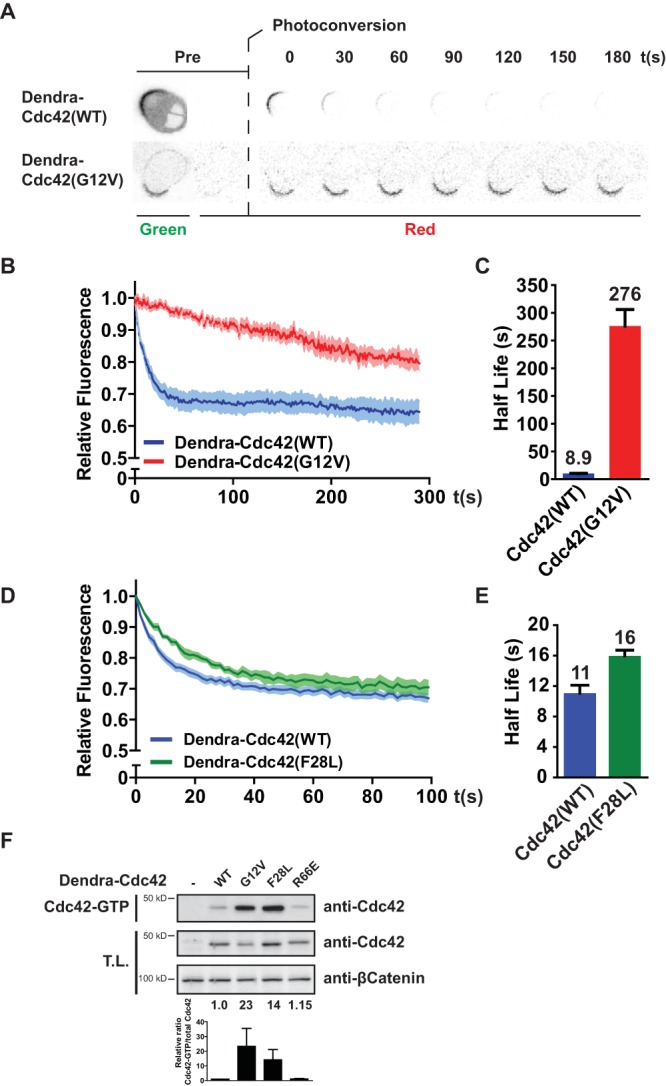
Apical mobility of Cdc42 is regulated in an activity-dependent manner. (A) Images from photoconversion experiments in polarized Ls174T:W4 cells expressing Dendra-Cdc42(WT) or (G12V). (B) Average normalized fluorescence decay traces after photoconversion of cells expressing Dendra-Cdc42(WT) (*n* = 11) and (G12V) (*n* = 14). Light-colored areas reflect standard errors of the means. (C) Eviction half-lives determined from average decay traces shown in panel B using curve fitting. Error bars represent the 95% confidence interval of the fit. (D) Average normalized fluorescence decay traces after photoconversion of cells expressing Dendra-Cdc42(WT) (*n* = 14) and Cdc42(F28L) (*n* = 8). Light-colored areas reflect standard errors of the means. (E) Eviction half-lives determined from average decay traces shown in panel D using curve fitting. Error bars represent the 95% confidence interval of the fit. (F) Cdc42-GTP pulldown of Dendra-Cdc42 WT, G12V, F28L, and R66E from HEK293T cell lysate. The bottom graph shows the quantification of relative Cdc42-GTP versus total Cdc42 levels. Error bars represent standard deviations in three experiments. T.L., total lysate.

Using this strategy, we compared the mobility of constitutively activated Dendra-tagged Cdc42 with a G12V substitution [Dendra-Cdc42(G12V)] with that of wild-type Cdc42 [Cdc42(WT)] and found a striking difference in mobilities: whereas wild-type Cdc42 is highly mobile at the apical plasma membrane, Cdc42(G12V) is relatively immobile, with an approximately 30-fold longer eviction half-life (8.9 s versus 276 s, respectively) (Fig. 1A to C). In contrast, the mobility of the fast-cycling Cdc42(F28L) mutant, which is more GTP loaded than the wild type, is similar to that of the wild type, indicating that the ability to cycle between active and inactive states allows high mobility (Fig. 1D to F). Since the immobilization of active Cdc42 is likely to drive the establishment of localized Cdc42 signaling, we set out to identify the molecular mechanisms responsible for Cdc42 immobilization upon GTP loading.

**FIG 2:**
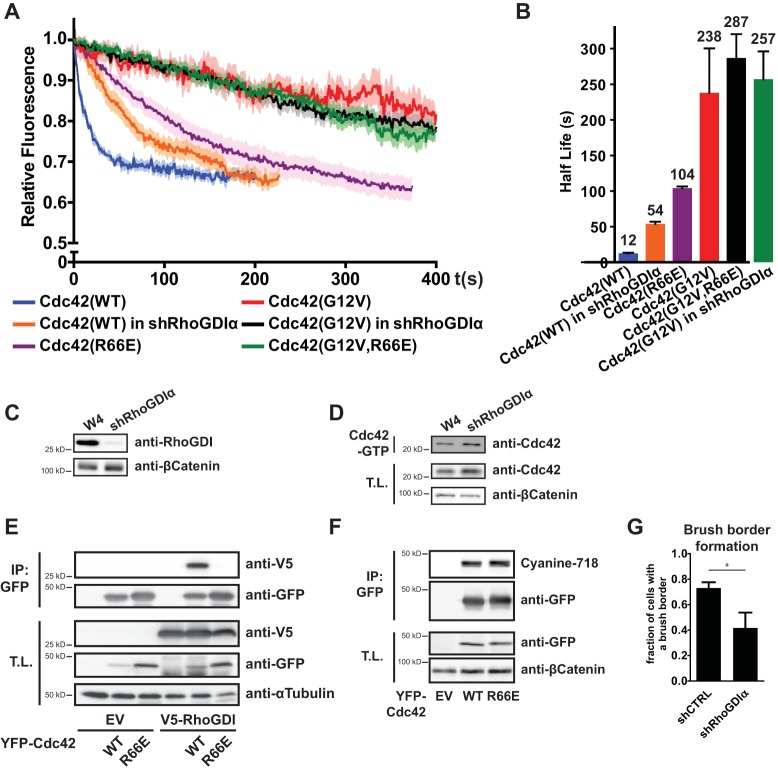
RhoGDI selectively increases mobility of inactive Cdc42. (A) Average normalized fluorescence decay traces of Dendra-Cdc42(WT) in W4 cells (blue line, *n* = 11) and W4:shRhoGDIα cells (orange line, *n* = 11), Dendra-Cdc42(R66E) in W4 cells (*n* = 12), Dendra-Cdc42(G12V) in W4 cells (red line, *n* = 16) and W4:shRhoGDIα cells (black line, *n* = 17), and Dendra-Cdc42(G12V, R66E) in W4 cells (*n* = 13). Light-colored areas reflect standard errors of the means. (B) Eviction half-lives determined from average decay traces shown in panel A using curve fitting. Error bars represent the 95% confidence interval of the fit. (C) Western blot of W4 and RhoGDIα-depleted W4 (shRhoGDIα) cell lysates, probed for RhoGDI and β-catenin. (D) Cdc42-GTP pulldown of endogenous Cdc42 from W4 and RhoGDIα-depleted W4 cell lysates. (E) Coimmunoprecipitation of YFP-Cdc42(WT) or YFP-Cdc42(R66E) with V5-RhoGDI in HEK293T cells. (F) Cyanine-718 labeling of geranylgeranylated YFP-Cdc42(WT) or YFP-Cdc42(R66E) in HEK293T cells. (G) Quantification of brush border formation in W4:shCTRL and W4:shRhoGDIα cells. Error bars indicate standard errors of the means (*n* = 3). *, *P* < 0.05, using an independent sample *t* test. IP, immunoprecipitation.

RhoGDIs bind small GTPases of the Rho family and facilitate their dissociation from the plasma membrane (17, 18). Although RhoGDI has similar affinities for Cdc42-GDP and Cdc42-GTP in solution, when Cdc42 is inserted in an artificial bilayer, RhoGDI has a higher affinity for Cdc42-GDP (18). Therefore, RhoGDIs may selectively increase the mobility of Cdc42-GDP. Indeed, in Ls174T:W4 cells with stable knockdown of RhoGDIα, the mobility of wild-type Cdc42 was reduced (eviction half-life of 53.8 s) (Fig. 2A to C). In RhoGDIα-depleted cells, no increase in total Cdc42 activity was observed, indicating that loss of RhoGDIα does not affect Cdc42 mobility by controlling Cdc42 activity (Fig. 2D). In addition, Cdc42(R66E), which is unable to interact with RhoGDIs, displays an even slower apical mobility (half-life of 104 s) (Fig. 2A, B, and E). Importantly, the R66E mutation does not affect the global GTP loading or posttranslational lipid modification of Cdc42 (Fig. 1F and 2F). The observed difference in half-lives between RhoGDIα knockdown and Cdc42(R66E) may be of technical origin: knockdown of RhoGDIα impairs brush border formation, and since mobility is assessed only in cells with a brush border, these cells are likely to have only moderate knockdown (Fig. 2G). Importantly, membrane dissociation by RhoGDI selectively acts on Cdc42-GDP as the mobility of constitutively active Cdc42(G12V) is not affected by introduction of the R66E mutation or RhoGDIα knockdown (Fig. 2A and B).

**FIG 3:**
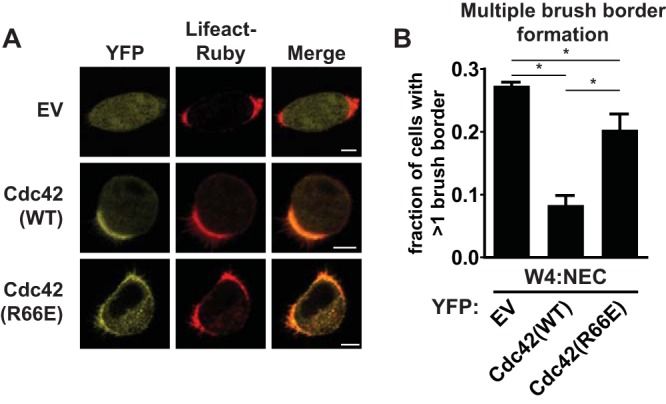
The interaction of Cdc42 with RhoGDI is required for the formation of a single brush border. (A) W4:NEC cells expressing the actin marker Lifeact-Ruby in combination with YFP (EV, empty vector), YFP-Cdc42(WT), or YFP-Cdc42(R66E). Scale bar, 5 μm. (B) Quantification of cells with multiple brush borders in W4:NEC cells expressing YFP, YFP-Cdc42(WT), or YFP-Cdc42(R66E). Error bars are standard errors of the means (*n* = 3). *, *P* < 0.05, using an independent sample *t* test.

To assess the functional contribution of RhoGDI-mediated membrane dissociation of Cdc42-GDP, we tested whether Cdc42(R66E) was able to restore singularity in apical membrane formation in Ls174T:W4 cells that have no endogenous Cdc42 (W4:NEC cells). We previously reported that these Cdc42 knockout cells retain the ability to form a brush border but are nonetheless unable to ensure that polarization is restricted to a single site (3). We find that the singularity defect in the W4:NEC cells is rescued by reintroduction of wild-type Cdc42, demonstrating that yellow fluorescent protein (YFP)-tagged Cdc42 is still functional (Fig. 3). Nonetheless, expression of YFP-Cdc42(R66E) could not restore singularity, indicating that the interaction with RhoGDIs is required to concentrate Cdc42 into a single cluster and is therefore required for Cdc42 function (Fig. 3).

**FIG 4:**
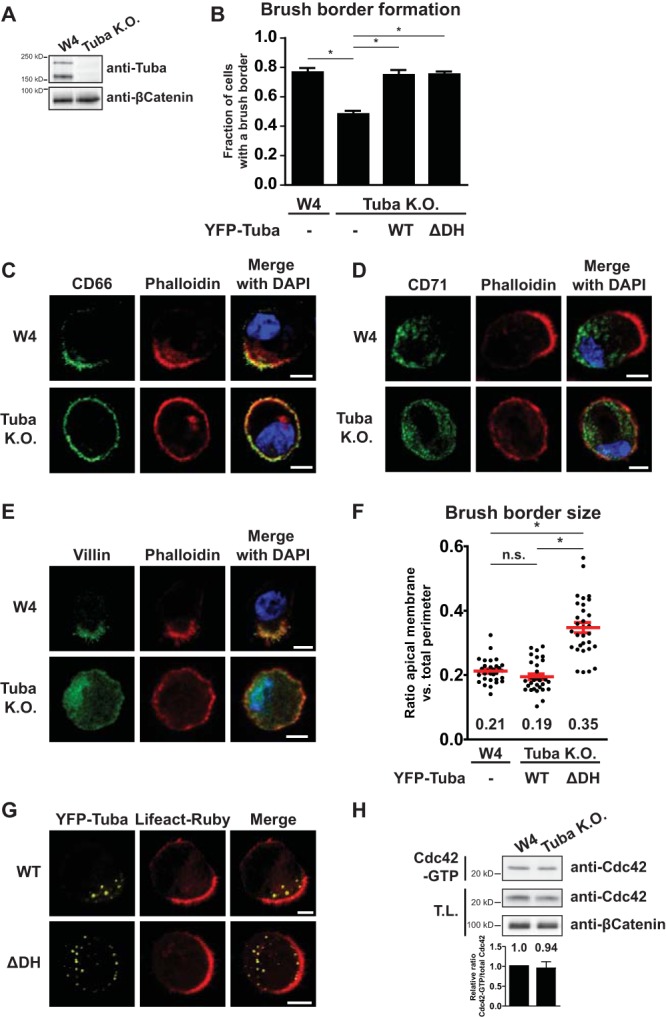
Tuba has a Cdc42-dependent and-independent function during enterocyte polarization. (A) Western blot of cell lysates of W4 cells and Tuba knockout (KO) W4 cells, probed for Tuba and β-catenin. (B) Quantification of brush border formation in W4 cells or Tuba knockout cells expressing Lifeact-Ruby alone or in combination with YFP-Tuba(WT) or YFP-Tuba(ΔDH). Error bars are standard errors of the means (*n* = 3). *, *P* < 0.05, using an independent sample *t* test. (C to E) Immunofluorescence of W4 or Tuba knockout cells stained for the apical membrane marker CD66, the basolateral marker CD71, and the brush border marker villin, as indicated. Scale bars, 5 μm. (F) Quantification of brush border size in W4 cells expressing Lifeact-Ruby and Tuba knockout cells expressing Lifeact-Ruby in combination with YFP-Tuba(WT) or YFP-Tuba(ΔDH). Red lines indicate the averages and standard errors of the means (*n* = 30 in 3 independent experiments). *, *P* < 0.05, using an independent sample *t* test; n.s., not significant (*P* > 0.05). (G) Representative images of Tuba knockout cells expressing Lifeact-Ruby and YFP-Tuba(WT) or YFP-Tuba(ΔDH). Scale bars, 5 μm. (H) Cdc42-GTP pulldown of endogenous Cdc42 from W4 and Tuba knockout W4 cell lysates.

Although RhoGDI-dependent membrane dissociation of Cdc42-GDP contributes to the high mobility of inactive Cdc42, it can only in part explain the immobilization of Cdc42 upon GTP loading. To find the mechanism that is responsible for the additional immobilization of Cdc42-GTP, we focused on the GEF for Cdc42, reasoning that RhoGEFs often act as a scaffold for the complexation of Cdc42-GTP with its effector and that this complexation could limit the mobility of activated Cdc42. Tuba (also known as DNMBP or ArhGEF36) is a Cdc42-specific GEF that has been implicated in regulating Cdc42 signaling in polarizing MDCK cysts and during zebrafish development (2, 19–22). We therefore tested whether Tuba, like Cdc42, is involved in apical membrane clustering in polarized Ls174T:W4 cells. Surprisingly, Tuba knockout cells showed a decreased ability to generate a brush border and to polarize, as judged by the distribution of apical (CD66), basolateral (CD71), and brush border (villin) markers (Fig. 4A to E). This phenotype cannot be attributed to loss of Cdc42 signaling because Cdc42 knockout cells are unaffected in their ability to form brush borders (3). Therefore, in order to specifically address the Cdc42-dependent effects of Tuba, we reintroduced either wild-type Tuba or a mutant lacking the GEF domain [Tuba(ΔDH)] in Tuba knockout cells. Reexpression of wild-type Tuba rescued the brush border formation defect and resulted in brush borders of normal size, excluding potential off-target effects of the CRISPR/Cas9 procedure used to generate the knockout cells (Fig. 4B, F, and G). In contrast, whereas expression of Tuba(ΔDH) was able to restore brush border formation, it resulted in the formation of brush borders that were larger in size, which is the phenotype associated with defective Cdc42 clustering in Ls174T:W4 cells (Fig. 4B, F, and G). This therefore demonstrates that Tuba, along with a Cdc42-independent function in cell polarization, is required for apical membrane clustering in polarized Ls174T:W4 cells. Similar to previous investigators, we do not find a large decrease in total Cdc42-GTP levels in Tuba knockout cells, suggesting that Tuba regulates a small fraction of cellular Cdc42 activity (Fig. 4H) (22, 23). Fluorescence resonance energy transfer (FRET)-based assays for Cdc42 activity were not feasible because the geometry of the brush border affected FRET measurements.

**FIG 5:**
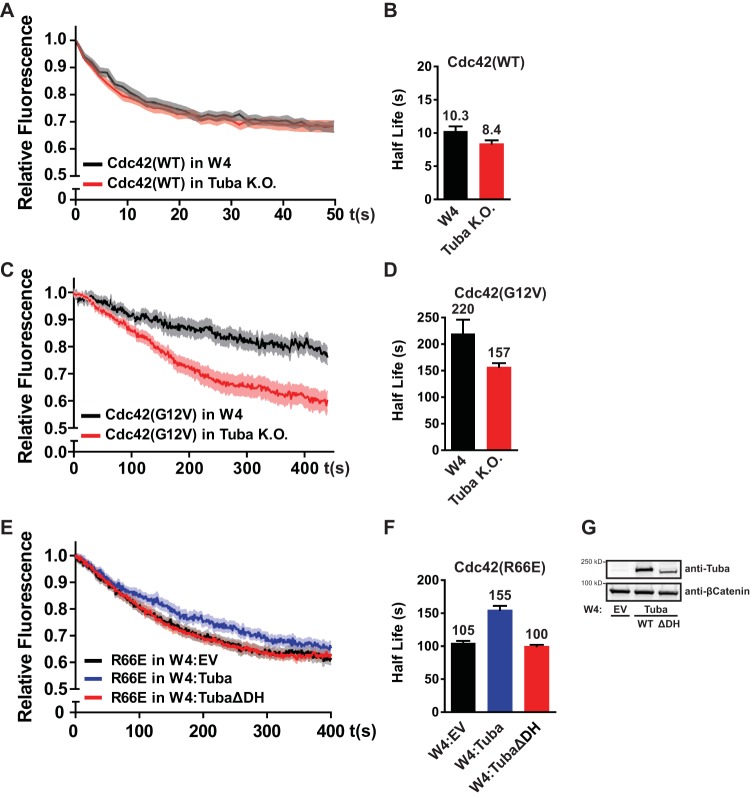
Tuba selectively immobilizes activated Cdc42 at the apical membrane. (A) Average normalized fluorescence decay traces of Dendra-Cdc42(WT) in W4 cells (*n* = 8) and in Tuba knockout cells (*n* = 11). (B) Eviction half-lives for Dendra-Cdc42(WT) in W4 and Tuba knockout cells determined from average decay traces shown in panel A using curve fitting. (C) Average normalized fluorescence decay traces of Dendra-Cdc42(G12V) in W4 cells (*n* = 14) and Tuba knockout cells (*n* = 16). (D) Eviction half-lives for Dendra-Cdc42(G12V) in W4 and Tuba knockout cells determined from average decay traces shown in panel C using curve fitting. (E) Average normalized fluorescence decay traces of Dendra-Cdc42(R66E) in W4 expressing an empty vector (EV; *n* = 20), in Tuba-overexpressing cells (*n* = 17), and in Tuba(ΔDH)-overexpressing cells (*n* = 20). (F) Eviction half-lives for Dendra-Cdc42(R66E) from average decay traces shown in panel E using curve fitting. Light areas in panels A, C, and E represent standard errors of the means. Error bars in panels B, D, and F represent the 95% confidence intervals of the fit. (G) Western blot of lysates of W4 cells expressing an empty vector(W4:EV), Tuba-overexpressing cells, and Tuba(ΔDH)-overexpressing cells probed for Tuba and β-catenin.

Next, we addressed whether Tuba contributes to the diffusive behavior of Cdc42 in polarized Ls174T:W4 cells. For this we determined the apical eviction half-life of wild-type Cdc42 and Cdc42(G12V) in polarized Ls174T:W4 cells and in the fraction of Tuba knockout cells that were polarized. We found that the mobility of wild-type Cdc42 is minimally affected by Tuba loss (10.3 s in Ls174T:W4 cells versus 8.4 s in Tuba knockout cells) (Fig. 5A and B). In contrast, Cdc42(G12V) was more rapidly lost from the apical membrane in Tuba knockout cells (220 s versus 157 s), demonstrating that Tuba selectively affects the mobility of active Cdc42 (Fig. 5C and D).

In order to test whether the effect of Tuba on Cdc42 mobility is dependent on Tuba's GEF activity toward Cdc42, we assessed the mobility of Cdc42 in Ls174T:W4 cells stably overexpressing either wild-type Tuba or catalytically inactive Tuba(ΔDH) (Fig. 5G). As any promiscuous Cdc42 GEF may decrease the mobility of wild-type Cdc42 by increasing the fraction of RhoGDI-resistant Cdc42-GTP, we used Cdc42(R66E) to address whether Tuba is able to immobilize Cdc42 in a RhoGDI-independent manner. Indeed, we find that overexpression of wild-type Tuba is able to decrease the mobility of Cdc42(R66E) at the apical membrane (Fig. 5E and F). This effect is dependent on the GEF activity of Tuba because overexpression of Tuba(ΔDH) did not lead to a similar reduction in mobility of Cdc42(R66E) (Fig. 5E and F). Therefore, these data demonstrate that Tuba selectively immobilizes activated Cdc42 and is thus required to enable differential mobility between GDP- and GTP-bound Cdc42.

**FIG 6:**
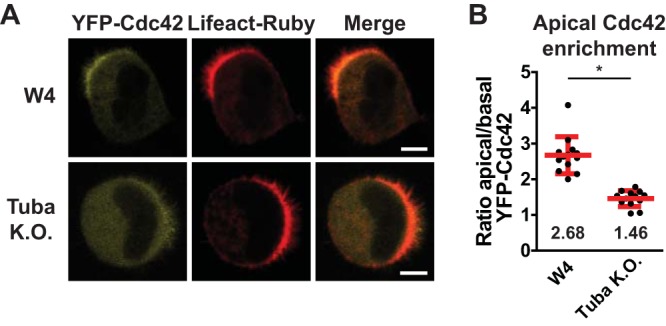
Tuba is required for Cdc42 enrichment at the apical plasma membrane. (A) Localization of YFP-Cdc42 in W4 and Tuba knockout cells in combination with Lifeact-Ruby. Scale bars, 5 μm. (B) Quantification of apical membrane enrichment of YFP-Cdc42 in polarized W4 or Tuba knockout cells. Red lines indicate the average and standard errors of the means (W4 cells, *n* = 13; Tuba knockout cells, *n* = 13; 3 independent experiments). *, *P* < 0.05, using an independent sample *t* test.

Finally, we tested whether Tuba-mediated immobilization of Cdc42 affects Cdc42 clustering at the apical membrane. In agreement with previous reports, we find that YFP-Cdc42 accumulates poorly at the apical membrane in polarized Tuba knockout cells, demonstrating a crucial role for Tuba in establishing localized Cdc42 signaling (Fig. 6) (20).

## DISCUSSION

Here, we show that during enterocyte polarization, the mobility of Cdc42 at the apical membrane is governed via a dual mechanism: first, RhoGDI-mediated membrane dissociation results in selective mobilization of inactive Cdc42, and second, activated Cdc42 is selectively immobilized by the Cdc42 GEF Tuba. The combined effect of this dual regulation is a switch-like behavior in Cdc42 mobility resulting in an over 30-fold decrease in Cdc42 mobility upon activation. This switch-like diffusive behavior ensures that upon GTP-loading, Cdc42 signaling remains spatially restricted. Therefore, this type of diffusive behavior may be important for many biological processes which involve localized Cdc42 signaling. Indeed, we show that interference with the mechanisms that enable differential Cdc42 mobility impedes local Cdc42 signaling during enterocyte polarization, resulting in an inability to cluster the apical membrane.

We show that the Cdc42-specific GEF Tuba is required for apical membrane clustering in a manner dependent on its catalytic GEF domain. However, we also notice that Tuba knockout cells have a reduced brush border formation capacity, a phenotype that is not dependent on Cdc42. We therefore reveal that Tuba has a dual function during cell polarization: one Cdc42-dependent function in apical membrane clustering and a second Cdc42-independent function in apical membrane formation. The notion that Tuba has a function in cell polarization independent of its GEF activity can explain recent findings in zebrafish where *tuba* knockdown results in a set of phenotypes that are not fully phenocopied by *cdc42* depletion (21). The molecular mechanism of Tuba's Cdc42-independent polarity function remains elusive, but it has been reported that Tuba binds many actin modulators that are important during cell polarization (24).

An important consequence of our finding that Tuba is able to specifically immobilize activated Cdc42 is that Tuba provides not only temporal control but also spatial control over Cdc42 signaling. By generating a pool of immobile, active Cdc42 molecules, Tuba enables clustering of active Cdc42. Indeed, we found that Cdc42 enrichment at the apical membrane is decreased in Tuba knockout cells, in agreement with previous findings in MDCK cysts (20). This poses the question of how Tuba itself becomes localized during cell polarization. We speculate that, analogous to yeast cell polarization, this may involve recruitment by activated Cdc42, thereby establishing a positive feedback mechanism for Cdc42 clustering. In support of this idea, we find that whereas localization of wild-type Tuba is mostly restricted to the apical domain, Tuba(ΔDH) localization is more scattered and not exclusively apical.

Although Tuba-mediated activation of Cdc42 enables differential mobility by both immobilizing Cdc42-GTP and by generating RhoGDI-resistant Cdc42-GTP, RhoGDI-mediated membrane dissociation of Cdc42-GDP is required for clustered Cdc42 signaling. We find that RhoGDI is required to maximize the differential mobility because whereas in the absence of RhoGDI Tuba allows only a 2.5-fold reduction in Cdc42 mobility upon GTP loading, the presence of RhoGDI results in a 30-fold reduction in mobility of Cdc42 upon activation. Therefore, our findings suggest that RhoGDI is required for Cdc42 clustering as it maximizes the difference between the mobilities of the inactive and active forms of Cdc42.

We speculate that Tuba-dependent immobilization of Cdc42-GTP results from the formation of a ternary complex composed of Tuba, Cdc42-GTP, and an effector protein. Neural Wiskott-Aldrich syndrome protein (NWASP) is a Cdc42 effector that is implicated in mediating Tuba- and Cdc42-dependent signaling during cell polarization (19, 22). We generated NWASP knockout Ls174T:W4 cells, but in these cells Cdc42 mobility was unaffected, and brush borders formed with normal size, indicating that Cdc42 likely engages a different effector for apical membrane clustering (data not shown).

Since Cdc42 is drastically immobilized upon GTP loading and because Cdc42-dependent symmetry breaking during yeast cell polarization occurs via a Turing-type activator-substrate mechanism, it will be interesting to further determine whether clustering of Cdc42-GTP during enterocyte polarization proceeds via a similar mechanism (14, 15). What makes this interesting from a biological perspective is that a Turing-type reaction-diffusion mechanism can mathematically account for complex biological phenomena, such as symmetry breaking and ensuring singularity in apical membrane formation (14, 15).

In summary, we show how differential diffusion of active and inactive Cdc42 is enabled by a molecular network comprised of a small GTPase, GEF, and GDI. Because of this simple network organization, it is expected that similar regulation of diffusive behavior occurs in a wide range of biological processes involving localized signaling by small GTPases.

## MATERIALS AND METHODS

### Cell culture and plasmids.

Ls174T:W4 cells were cultured in RPMI 1640 medium (Sigma) supplemented with 10% fetal bovine serums (FBS; Lonza) and antibiotics. HEK293T cells were cultured in Dulbecco's modified Eagle's medium (DMEM) (Sigma) containing 10% FBS (Sigma) and antibiotics. Polarization was induced by culturing Ls174T:W4 cells in medium containing 1 μg/ml doxycycline (Sigma) for at least 16 h. For transient expression of DNA constructs, cells were transfected using XtremeGene9 (Roche) according to the manufacturer's guidelines.

N-terminally tagged Dendra-Cdc42 constructs were generated by introducing a Cdc42 PCR product in pDendra2-C2 using In-Fusion (Clontech). G12V and R66E mutations were introduced by site-directed mutagenesis. pcDNA-HA-Tuba (where HA is hemagglutinin) was provided by Pietro di Camilli. This construct, encoding the canonical long 177-kDa isoform of Tuba, served as a template to generate full-length Tuba or Tuba(ΔDH) entry clones which were subsequently N-terminally tagged with YFP using Gateway cloning (Invitrogen). To generate Tuba(ΔDH), in which amino acids 799 to 982 were deleted, two Tuba PCR fragments were generated (one upstream of the DH domain and one downstream) and assembled using In-Fusion. MiniTol2 constructs for stable overexpression of untagged Tuba were generated by introducing full-length or Tuba(ΔDH) PCR products in miniTol2-EF1α-MCS-PGK-PuroR using In-Fusion. pCMV-transposase was provided by Stephen Ekker, and Lifeact-Ruby was provided by R. Wedlich-Soldner.

### Antibodies.

The following antibodies were used for immunofluorescence: mouse anti-CD66 (1:500; BD Biosciences) and mouse anti-CD71 (1:1,000) (H68.4, Life Technologies,). For Western blotting the following antibodies were used: rabbit anti-RhoGDI (1:5,000; Millipore), rabbit anti-dynamin binding protein (anti-DNMBP) (1:2,000; Sigma), mouse anti-β-catenin (1:5,000; BD Biosciences), mouse anti-green fluorescent protein (anti-GFP; 1:5,000) (clones 7.1 and 13.1; Roche), mouse anti-V5 (1:5,000; Invitrogen), and mouse anti-α-tubulin (1:5,000; Calbiochem).

### Generation of RhoGDIα knockdown, Tuba knockout, and Tuba overexpression cell lines.

For stable knockdown of RhoGDIα, Ls174T:W4 cells were infected for two successive days with lentiviral short hairpin RNA (shRNA) constructs (yielding W4:shRhoGDIα cells) (Mission library; Sigma) and subsequently selected for puromycin resistance. To stably deplete RhoGDIα, five pooled short hairpins were used with the following targeting sequences: shRNA 1, 5′-AGCTTCAAGAAGCAGTCGTTT-3′; shRNA 2, 5′-CGTCTAACCATGATGCCTTAA-3′; shRNA 3, 5′-CAAGATTGACAAGACTGACTA-3′; shRNA 4, 5′-CCGCTTCACAGACGACGACAA-3′; shRNA 5, 5′-GACTACATGGTAGGCAGCTAT-3′.

Tuba knockout Ls174T:W4 cells were generated using CRISPR/Cas9-mediated gene disruption as previously reported (3). Briefly, Ls174T:W4 cells were transfected with pSpCas9(BB)-2A-GFP (PX458), encoding a short guide RNA (sgRNA) (AAGAGACTACATTCGGGATC) targeting the exon encoding the DH domain of Tuba. GFP-positive cells were sorted and clonally expanded. Genomic DNA of candidate clones was sequenced, and absence of Tuba protein was confirmed by Western blotting.

For stable overexpression of untagged Tuba, Ls174T:W4 cells were transiently cotransfected with miniTol2-Tuba constructs and Tol2 transposase in a 2:1 ratio. Three days after transfection, cells were selected for puromycin resistance and continuously cultured in the presence of puromycin (10 μg/ml). Monoclonal cell lines were established by serial dilution, and the presence of Tuba was assessed by Western blotting.

### Live-cell imaging.

Two days after transfection, Ls174T:W4 cells were trypsinized and plated onto glass-bottom dishes (WillCo Wells) in the presence of doxycycline. Before imaging, medium was replaced with HEPES-buffered (pH 7.4) Leibovitz's L-15 medium (Invitrogen). Cells were imaged at 37°C using an Axioskop2 LSM510 scanning confocal microscope (Zeiss) with a 63× oil objective (Plan Apochromat; numerical aperture [NA], 1.4) using Zen image acquisition software. To determine apical membrane size, the fraction of cell membrane that was covered with microvilli was determined using ImageJ software. Average apical membrane sizes were compared using an independent samples *t* test in SPSS with a *P* value of <0.05 as a cutoff for significance. Apical enrichment of YFP-Cdc42 was quantified by making a line plot through the apical and basal membrane using ImageJ and subsequently determining the ratio between average apical and basal membrane pixel intensities. Average enrichment ratios were compared using an independent samples *t* test in SPSS with a *P* value of <0.05 as a cutoff for significance.

### Apical membrane eviction rates.

To determine apical mobility of Cdc42, cells were transfected with Dendra-Cdc42. After 2 days, cells were split and seeded onto glass-bottom dishes in the presence of doxycycline. Before imaging, medium was replaced with HEPES-buffered (pH 7.4) Leibovitz's L-15 medium. Photoconversion experiments were performed on a Leica SP8x microscope equipped with a temperature- and CO_2_-controlled chamber using a 63× oil objective (HC PL APO 63×/1.40) with Leica LAS AF image acquisition software. Local Dendra photoconversion in the brush border was done using a pulse of 405-nm laser light. Cells were subsequently imaged at a frame rate of 1.5 s/frame. To determine the average decay rate, the ratio of the average intensity of red signal in the brush border to the average intensity of red signal in the whole cell was determined. Moving regions of interest were used to correct for cell movements during imaging. For each cell, ratios were normalized, and traces were averaged to generate an average fluorescence decay trace. Using Matlab software, these curves were fitted with the following general formula: *f*(*x*) = *ae*^(−*bx*)^ + *c*. An average half-life was determined from the fitted curve and expressed with the 95% confidence interval (CI) of the fit.

### Immunofluorescence.

Cells were seeded on glass coverslips in the presence of doxycycline and subsequently fixed in 4% methanol-free formaldehyde (Electron Microscopy Sciences) for 30 min, permeabilized in 0.1% Triton X-100 in phosphate-buffered saline (PBS) for 10 min, and blocked in 2% bovine serum albumin (BSA) in PBS for 1 h. Slides were then incubated with primary antibody for at least 16 h, washed three times with PBS, and incubated with Alexa Fluor 488-coupled secondary antibody in the presence of 4′,6′-diamidino-2-phenylindole (DAPI) and phalloidin-Alexa Fluor 568 (ThermoFisher) for at least 4 h. After PBS washes, slides were mounted and imaged using an Axioskop2 LSM510 scanning confocal microscope (Zeiss) equipped with a 63× oil objective (Plan Apochromat; NA of 1.4) using Zen image acquisition software.

### Immunoprecipitation.

Two days after transfection, HEK293T cells were lysed in RAL buffer (1% Nonidet P-40 substitute, 10% glycerol, 50 mM Tris-HCl, pH 7.4, 2.0 mM MgCl_2_, 200 mM NaCl, protease and phosphatase inhibitors) on ice. Lysates were cleared by centrifugation and incubated with GFP-binding protein (GBP)–agarose beads for 2 h at 4°C with rotation. Beads were washed with RAL buffer three times and eluted in sample buffer.

Cdc42-GTP was precipitated using PAK1-PBD-agarose beads (Millipore) according to the manufacturer's instructions. To assess geranylgeranylation, transfected cells were incubated in medium containing 50 μM geranylgeranyl alcohol azide (Life Technologies) for 24 h. Labeled YFP-Cdc42 was precipitated from cleared lysates and functionalized with alkyne cyanine-718 (Sigma) using copper(I)-catalyzed azide-alkyne cycloaddition. For this, beads were resuspended in 94 μl of PBS, and 6 μl of click reaction mix was added, containing 10 μM alkyne cyanine-718, 1 mM tris(2-carboxyethyl)phosphine (TCEP), 100 μM tris[(1-benzyl-1H-1,2,3-triazol-4-yl)methyl]amine (TBTA), and 1 mM CuSO_4_. Beads were incubated for 2 h at room temperature with rotation, washed three times with 1% Nonidet P-40 substitute in PBS, and eluted in sample buffer. Samples were subjected to SDS-PAGE, and cyanine-718 labeling was detected by in-gel fluorescence imaging using a Li-Cor Odyssey system.
